# Connecticut providers knowledge and attitudes towards use of extreme risk protection orders

**DOI:** 10.1186/s40621-025-00565-1

**Published:** 2025-03-19

**Authors:** Nishant Pandya, James Dodington, Joshua Jacob, Sarah Raskin

**Affiliations:** 1 Children’s Hospital of Philadelphia, Pennsylvania 4435 Sansom St Apt 2, Philadelphia, PA 19104 USA; 2 Pediatrics (Emergency Medicine), Injury and Violence Prevention at Yale School of Medicine, 1 Park Street, New Haven, CT 06504 USA; 3 Trinity College Department of Psychology and Neuroscience Program, 300 Summit Street, Hartford, CT 06106 USA

## Abstract

**Background:**

Extreme Risk Protection Orders (ERPOs) are a legislative tool that temporarily restrict firearm access and purchasing ability in patients at risk for harm. Data from four states with ERPO legislation, including Connecticut, estimates 17 to 23 filed ERPOs can prevent 1 suicide. Connecticut medical providers are permitted to independently file an ERPO directly to the courthouse. This survey assesses provider knowledge and attitudes towards use of ERPOs.

**Methods:**

This study electronically surveyed providers from six hospitals regarding their current knowledge of the Connecticut ERPO law, perceived barriers to the use of the law and procedures that might make use more likely.

**Results:**

114 providers completed the survey in 2022. 66 (57.8%) providers encountered at least 1 patient per year at risk for suicide with firearm access. Only 2 (1.7%) providers had ever initiated an ERPO, but both found it extremely helpful. Only 1 provider was extremely familiar with ERPO while 91 (78.9%) were not familiar. Barriers to using ERPO include negatively impacting the patient relationship, and not enough time to call and follow up. ERPO specific training, and trained on-site coordinators to help file and follow through were ways to encourage to ERPO utilization.

**Conclusion:**

The majority of providers encounter at least one patient annually who may benefit from ERPO utilization. However, providers are largely unfamiliar with ERPO and the filing process. Time cost is the greatest barrier to its utilization. Provider training and trained coordinators to process ERPO were the two most requested supports to encourage providers to initiate ERPOs.

**Supplementary Information:**

The online version contains supplementary material available at 10.1186/s40621-025-00565-1.

## Introduction

In 2022, there were 27,032 firearm-related suicides in the United States. Suicide was the 11th leading cause of death in the United States that year and suicide by firearms were responsible for nearly 55% of suicides [1, 2]. Extreme risk protection orders (ERPO) are one legislative tool that can help address suicides by firearm. These laws create a legal process by which a court can order firearm removal or restrict firearm purchase from individuals who are deemed to be at risk of imminent harm to themselves or others, and when other alternatives for preventing access to the firearms have been exhausted. They are aimed at reducing firearm violence including suicide and interpersonal violence.

Connecticut passed the United States’ first state-wide ERPO law in 1999 in response to a mass shooting the previous year. Despite active concerns about the shooter’s mental health, there had been no legal means for the police to intervene [3]. The original Connecticut ERPO law permitted law enforcement to request permission from a judge to temporarily remove and prohibit the future acquisition of firearms for those deemed at “extreme risk” to themselves or others.

As of August 2025, 21 additional states, California, Colorado, Delaware, Florida, Hawaii, Illinois, Indiana, Maryland, Massachusetts, Michigan, Nevada, New Mexico, New Jersey, New Mexico, New York, Oregon, Rhode Island, Vermont, Virginia, Washington, and the District of Columbia in the US have enacted ERPO laws [4]*.*

More than 2,600 ERPOs were filed in Connecticut between its creation in 1999 and 2022. Suicidality or risk for self-injury as the most common reason for firearm seizure [5]. Connecticut experienced an increase in ERPO utilization and firearm seizure following the high-profile Virginia Tech campus shooting, which was estimated to reduce firearm based suicides by 13.7% between 2007–2013 [3]. Looking at data from California, Connecticut, Maryland, and Washington, it is estimated 1 suicide death is prevented per every 17–23 firearm removals [6, 7].

Historically, the ERPO utilization process has been primarily initiated by law enforcement. An ERPO law was enacted in Washington state in 2016. Looking specifically at King County, the state’s most populous country, 75 ERPO petitions were filed in the 12 months following the law’s implementation to limit the purchase or possession of firearms by individuals. Despite family members, intimate partners, and household members being able to file an ERPO in this state, nearly all petitions were filed directly by law enforcement after being informed by family members that the individual was deemed at risk of suicide or harm to others [8]. California implemented ERPO laws in 2016. Similar to King County, over 90% of the 1076 petitions between 2016–2019 petitions were filed by law enforcement as opposed to family and household members. Each year, the total number of petitions filed did increase but great variability exists in the social demographics of the individuals that petitions were filed against and citizen comfort with initiating an ERPO petition [9].

In 2022, Connecticut expanded its ERPO law to allow medical professionals and family members to petition directly to a law enforcement officer or a court to initiate a risk order protection investigation. They join Colorado, Hawaii, Maryland, Michigan, New York, and the District of Columbia as states where medical providers can file ERPOs. This change allows for family members and medical providers to file petitions directly to the court. This petition, along with a judge’s order, would place the individual’s name into the National Instant Criminal Background Check System to prevent firearm purchase and ask the police to conduct an investigation to assess risk of harm. Compared to the 96 warrants issued in Connecticut in the first six months of 2022, an estimated 418 ERPO warrants were granted between the new law’s implementation in June through November 2022 [10]. The demographics of those initiating ERPO before and after the law change is unclear. However, this change has created a path for family members and medical providers to file petitions directly to the court rather than asking police officers to petition a judge to restrict firearm access [10].

With the ability to file an ERPO warrant, Connecticut medical providers are in a privileged position to recognize periods of crisis, changes in mental health, or warning signs that an individual is at greater risk to themselves or others [11].

The United States Surgeon General, American Medical Association, American Academy of Pediatrics, Psychological Society, and American Academy of Family Physicians all recognize firearm violence as a public health crisis [9, 12]. ERPO laws are effective tools healthcare providers can use to help reduce firearm-related suicide and other deaths.

In a survey in the state of Maryland, healthcare providers in the departments of psychiatry, pediatrics, and emergency medicine were sent a survey to determine what they knew about the state’s ERPO law and what the barriers and facilitators might be towards utilization [13]. The current study is designed to replicate and extend this Maryland study with changes made due to the differences between the laws in these states.

This target population for this survey include providers whose clinical work includes discussing mental health and suicidality. While other specialties may discuss these topics, primary care and mental health providers were the target population for this survey. In addition, we will ask questions about the subjective data that people currently find important in determining the use of ERPO laws so that we can analyze the likelihood that the law is being used in a just and fair way. Swanson (2020) has pointed out ways in which individual racial bias may play a role in subjective decisions to invoke the law. Finally, we included questions about whether a history of psychiatric or neurologic diagnosis impacts a providers’ decision to invoke ERPO restrictions. Having a mental health history is not a predictor of violence towards others [14]. However, psychiatric diagnoses can be comorbidities for patients with increased risk for suicide. [15].

Given the opportunity to reduce firearm-related suicide, it would be beneficial to better understand Connecticut healthcare providers’ attitudes towards ERPO laws and their perceived barriers to using them. This study surveyed medical providers in Connecticut in private practice and at four hospitals to assess knowledge, barriers, and facilitators to utilizing current ERPO laws in their clinical practice.

## Methods

### Participants

**Fig. 1:**
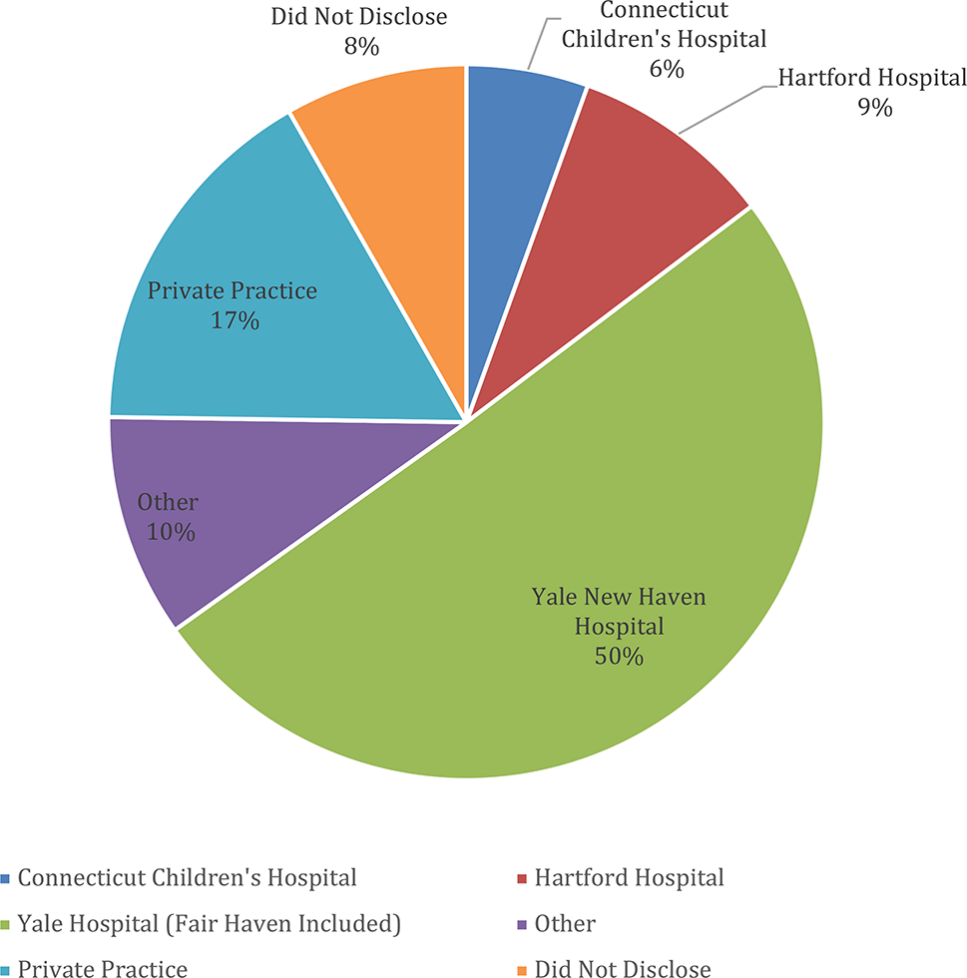
Home institution of each provider respondent

A total of 114 individuals responded to our survey. Detailed information about the type of practice, area of specialty, and practice location can be seen in Fig. 1.

### Materials

This survey contained 16 questions. After collecting demographic information, a series of Likert scale and multiple-choice questions were asked to assess provider knowledge and attitudes towards ERPO utilization along with information regarding clinical scenarios related to mental health and suicide counseling. To assess provider knowledge of ERPO, a brief summary of the Connecticut ERPO law and the process to utilize ERPO was provided in the beginning of the survey. The full survey can be viewed in Appendix I. It took approximately 10 min to complete.

### Procedure

The study was approved by the Institutional Review Board at Trinity College. The survey was created and distributed via Qualtrics software (Qualtrics, 2020). A survey link was sent to all members of the Connecticut Psychological Association via a listserv containing 350 individuals, and via 642 individual emails to members of the department listservs of psychiatry, internal medicine, and pediatrics at four regional hospitals (Yale New Haven Hospital, Hartford Hospital, Connecticut Children’s Medical Center, and Trinity Health of New England).

This survey was given to clinical providers whose patient facing work may involve mental health and suicidality. While other specialties may discuss these topics, primary care providers and mental health providers were the target population. Despite this targeted distribution, we did receive responses from providers in other departments and chose to keep them in the results. Participants had the option of receiving a $10 gift card for their participation. All surveys were completed in 2022.

## Results

Descriptive data were collected for all survey questions.

### Provider background

Area of practice for the respondents include providers specializing in pediatrics (37%), psychology (21%), psychiatry (15%), internal medicine (4%), emergency medicine (1%), gastroenterology (1%), OBGYN (1%) and undisclosed (25%).

### Awareness of ERPO and policies

**Fig. 2:**
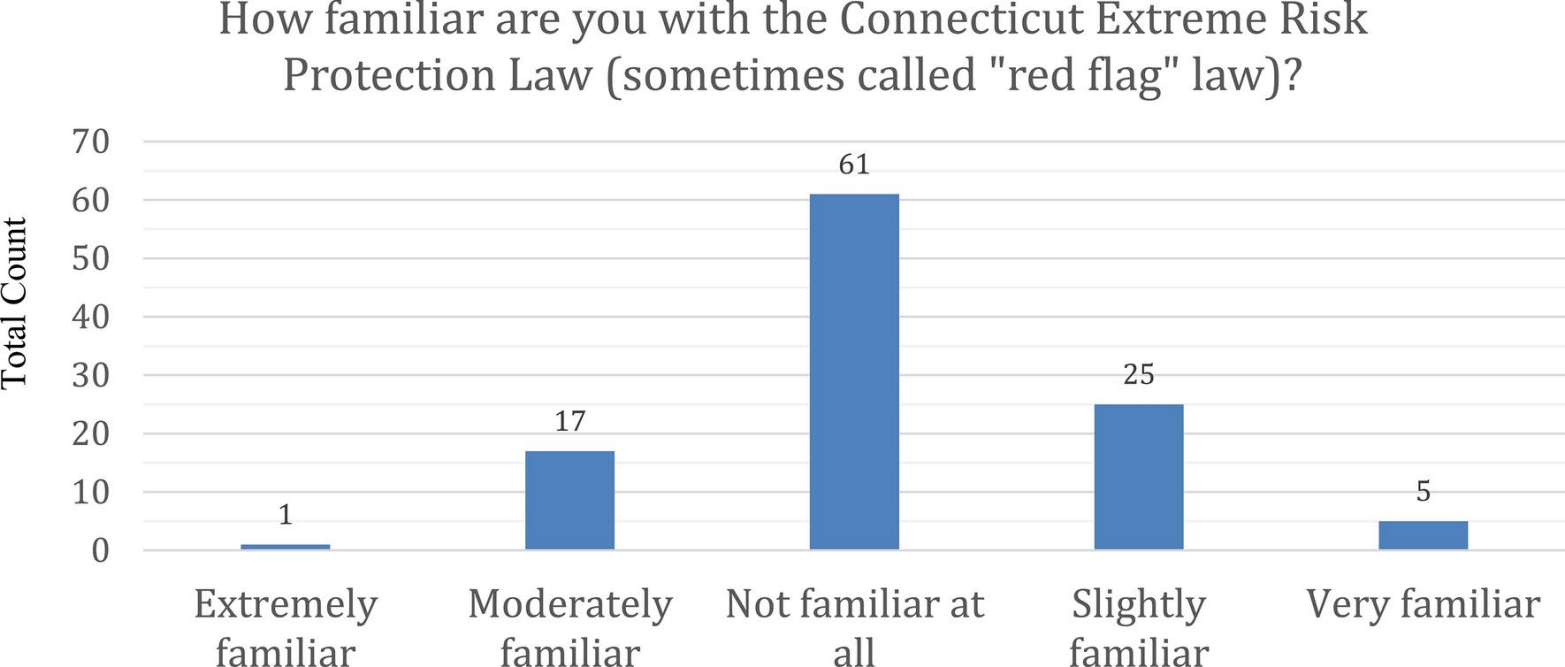
Provider familiarity with connecticut extreme risk protection law

When asked how familiar the participants were with the Connecticut ERPO law, only one (< 1%) said they were extremely familiar and five (4%) said they were very familiar. Seventeen (15%) were moderately familiar and 25 (22%) slightly familiar. Sixty-one (51% of respondents) reported that they were not at all familiar as seen in Fig. 2. None (0%) of the respondents were familiar with any specific policies or procedures surrounding the ERPO law at their institution.

### Potential need to use an ERPO

When asked “How often do you estimate you encounter a patient or client that is at extreme risk of violence, or suicide, has access to firearms, and who you would consider for an ERPO?” One (< 1%) respondent said daily, one (< 1%) weekly, and four (4%) monthly. Sixty (55%) respondents said a few times per year and 43 (39%) said never. Only two (2%) respondents had ever filed an ERPO petition for a patient at risk. Both respondents reported the act of filing an ERPO helpful for their patient.

### Current practice

**Fig. 3:**
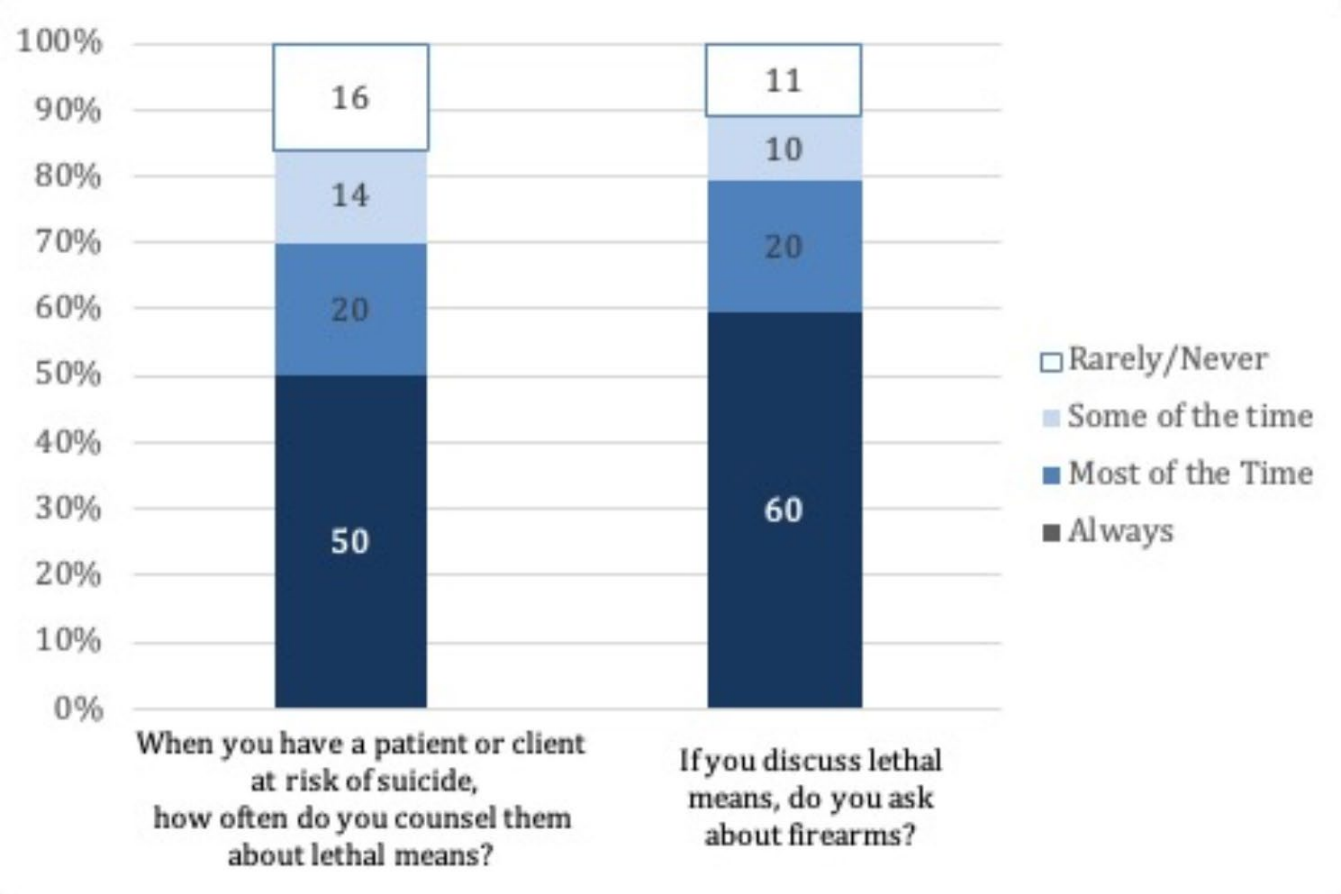
Percentage of providers counseling on lethal means to patients at risk for suicide

As shown in Fig. 3, 70% of respondents reported that they counsel those at risk of suicide about lethal means always or most of the time. Of those who do discuss lethal means, 80% or respondents discuss access to firearms always or most of the time.

### Barriers to using ERPO

The most cited barriers reported to using ERPO were concerns that it might negatively affect their relationship with the patient (26%), not enough time to make the call and follow up (20%), and not feeling comfortable bringing the police into patient care (26%). 24% of respondents marked “other” and most of the comments indicated that this was a lack of knowledge in how to file a report. When asked whether the ability to file an ERPO petition without directly involving the police would change their likelihood to file an ERPO, 26 (24%) respondents said they would be more likely to, 52 (45%) respondents said there would be no change, and 15 (13%) respondents said they would be less likely to file a petition.

### Suggestions to make it more likely to file an ERPO

Respondents were asked to give the most important way that systems could be changed to make it more likely for them to file an ERPO. The largest number of respondents (83%) felt they needed training. Having a trained coordinator at their institution to help with filing and follow through was the second most requested support by 68% of respondents. In addition, respondents would like to have consultation with a legal expert (45%) or a specific internal policy at their institution (47%).

### Impact of specific diagnoses

**Fig. 4:**
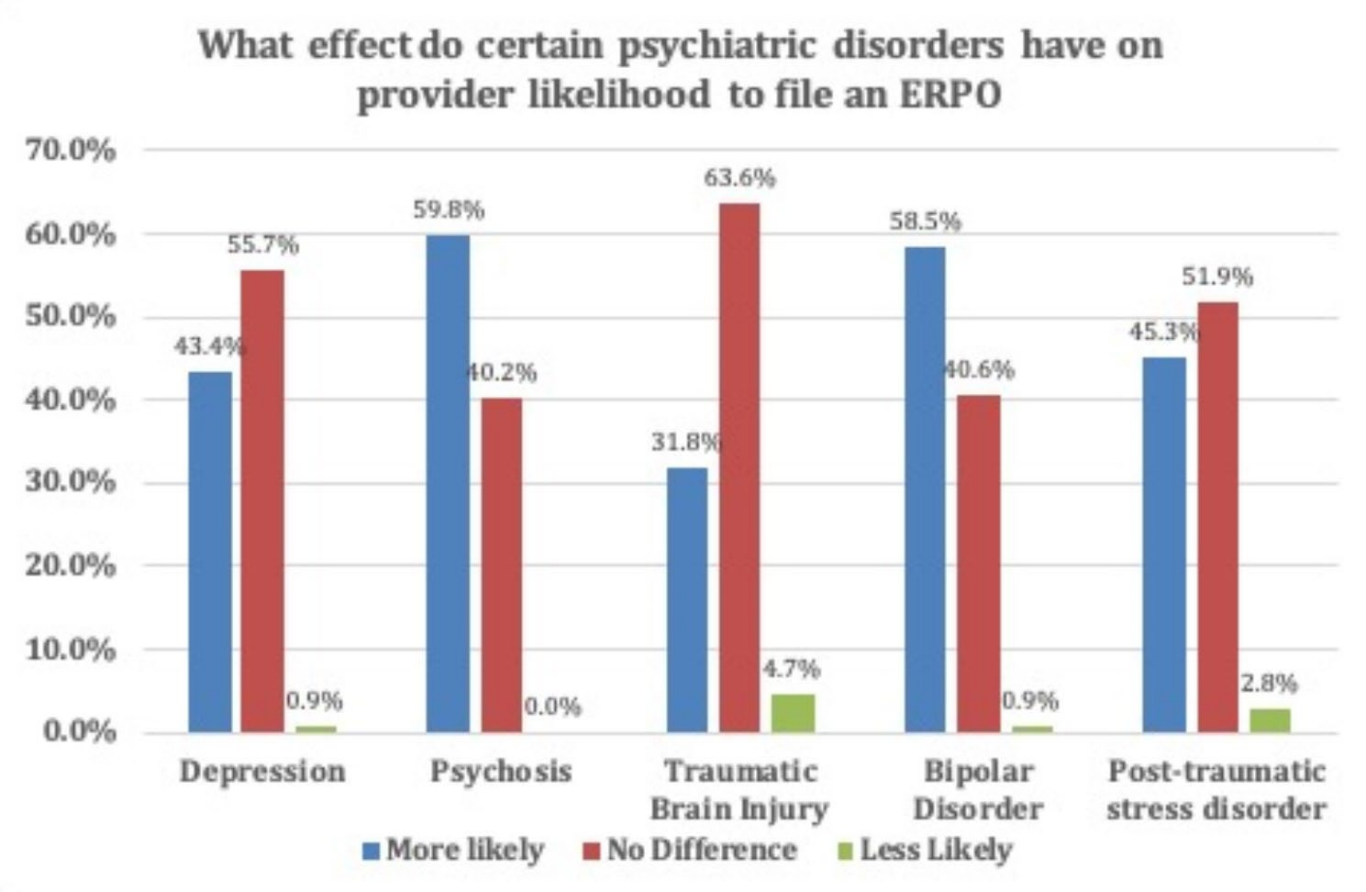
Effect of certain psychiatric disorders on provider likelihood to file an ERPO

The survey asked whether specific psychiatric disorders would make the provider more likely, less likely, or have no difference on the provider’s decision to file ERPO. The diagnoses included depression, psychosis, traumatic brain injury, bipolar disorder, and post-traumatic stress disorder. Providers did report being more likely to file ERPO for each diagnosis individually. The strongest increase in likelihood to file an ERPO was seen for patients with psychosis (59.8%) and bipolar disorder (58.5%). Those data can be seen in Fig. 4.

## Discussion

Of the surveyed providers, only two had ever filed ERPOs and 51% of providers were not familiar at all with Connecticut’s ERPO legislation prior to this survey. Connecticut was the first state to introduce ERPO in 1999 but only expanded the ability for medical providers to directly petition to state courthouses in 2022. While the ability to file an ERPO petition directly to the court is a recent change, the unfamiliarity with ERPO demonstrates a need to increase awareness among Connecticut medical providers. This tool has demonstrated the ability to reduce firearm suicides by temporarily removing and restricting the ability to purchase firearms. A 2019 study that assessed Maryland providers knowledge ERPOs revealed 71.7% of providers were not at all familiar with their states’ ERPO legislation [13]. The decrease in providers completely unfamiliar with ERPO may be due to increased awareness of legislation between 2019–2022 and increased discussion of ERPO in Connecticut following the expansion of the law in 2022.

Of surveyed providers, 61% said they see at least one patient annually who might benefit from ERPO. In the Maryland study, 92.4% of providers stated seeing at least one patient they would consider utilizing ERPO for [13]. Of note, that survey population only included only Psychiatrist, Pediatricians, and Emergency medicine physicians.

The number of patients who may benefit from ERPO may be underreported in our sample. The majority of providers are unaware of this legislation. Therefore, providers may not recognize clinical scenarios in which ERPOs may be appropriate to utilize. Additionally, providers are not guaranteed know whether a patient at risk for suicide as firearms in their home. Of note, over 30% of providers reported not regularly discussing lethal means when counseling for suicide. Of those who did such counseling, only 60% always asked about access to firearms. This gap may be attributed to lack of time or discomfort with counseling about firearm access and safely addressing all patient needs in the outpatient setting for both pediatric and adult primary care providers [16–18]. Often due to time barriers, it is not feasible to screen every single patient at every visit. Lack of time was the most frequently cited barrier to utilizing ERPO amongst the surveyed providers. Time was also cited as the largest barrier towards ERPO utilization in a series of 13 semi structured interviews [19].

Increased ERPO utilization can be a tool to reduce firearm suicides in Connecticut. Knowledge of procedure and time to file and follow up on ERPO are the two most commonly cited barriers cited by the surveyed providers. Institutions can support providers by investing in ERPO specific training, hiring trained coordinators, and providing legal counseling. These requests suggest a desire to learn more and engage with ERPO, but logistical, knowledge, and time barriers remain limiting factors. Even with such investment, respondents expressed concern of harming their relationships with patients and actively involving law enforcement into patient care. A large survey study in Washington state similarly found barriers including the feeling of ERPO falling outside of provider professional scope, concern for patient-provider relationship, concern for personal safety, and institutional barriers including lack of support staff [20]. Increasing knowledge of the ERPO petition filing and subsequent steps along with increased institutional support to appropriately screen, file, and follow up on ERPO would help support providers utilize ERPOs in their workflow.

ERPO, depression, bipolar disorder, PTSD, traumatic brain injury and psychosis were diagnoses that increased provider likelihood to utilize ERPO for a patient compared to baseline. These diagnoses do not put patients at increased risk of violence towards others. Patients with mental health disorders are at greater risk of being a victim of violence than committing violence [21]. These questions assessed what impact, if any, these diagnoses had on provider’s decisions to utilize ERPO given certain mental health conditions are associated with increased risk for suicide [15]. Provider education focused on recognizing the relationships between mental health disorders, risk of self-harm, and suicidality would be an important part of ERPO training. Patients with these diagnoses may better be served and screened for ERPO more regularly as on the one hand their risk for self-harm may be higher than the general population and on the other it would not be appropriate to assume that without adequate screening.

Limitations of this study include low survey participation rate and varied surveyed population. This survey was distributed via department email list-servs, but only received a 11.4% response rate. The survey was self-administered with an incentive to complete. The low response rate does increase risk of non-response bias. Additionally, the surveyed population includes providers from different medical specialties. While these providers all do have the potential to see patients who may benefit from utilization of ERPO, there can be differences in specific specialties the survey does not account for. Additionally, 25% of respondents did not disclose their medical specialty.

## Conclusion

This study surveyed medical providers in Connecticut assessing their current knowledge of the Connecticut ERPO law, perceived barriers to the use of the law and procedures to facilitate its use. The majority of surveyed Connecticut providers are unaware of ERPO and less than 2% had any experience utilizing this legislative tool as individuals or within their respective institutions. Despite their lack of familiarity, medical providers do encounter patients with risk for suicide with access to firearm in their clinical practice who may benefit from ERPO. Additionally, the majority of providers do appropriately counsel for and screen for lethal means and firearm access in patients at risk for suicide. Providing institutional support with ERPO specific training and hiring support staff to facilitate ERPO along are ways institutions can support providers to utilize this tool to help prevent suicide by firearm.

## Supplementary Information

Additional file1

## Data Availability

No datasets were generated or analysed during the current study.
