# Supplementary material for: Connecticut providers knowledge and attitudes towards use of extreme risk protection orders
Source: Inj Epidemiol. 2025 Mar 19;12:17. doi: 10.1186/s40621-025-00565-1 (PMC11921588; doi:10.1186/s40621-025-00565-1)
Supplement: Supplementary file 1 — Additional file1 [file 40621_2025_565_MOESM1_ESM.pdf]

Default Question Block

You are being asked to participate in a research study on the current incentives and barriers to using the Connecticut Extreme Risk Protection Order law. This study is being conducted in collaboration with Trinity College, Connecticut Children's Medical Center, Yale New Haven Hospital. You are being asked to participate because you are a health care provider. This survey should take no more than ten minutes. There are no risks to you in participating. There are no direct benefits to you in participating although you will be helping us understand more about how to prevent gun violence in the state of Connecticut. All of your responses will be strictly confidential and your name will not be associated with any of your responses. The original survey which will contain your email address will be kept in a password protected data file and will be destroyed six years after the survey is completed.

If you have any questions or concerns you may reach out to Sarah Raskin sarah.raskin@trincoll.edu or the Trinity College IRB at IRB@trincoll.edu.

I understand what is expected of me and agree to participate.

× SIGN HERE

clear

My primary affiliation is: (department/institution)

My role at this institution is

For how many years have you practiced in this specialty?

How familiar are you with the Connecticut Extreme Risk Protection Law (sometimes called "red flag" law)?

Extremely familiar

Very familiar

Moderately familiar

Slightly familiar

Not familiar at all

As a reminder:

Connecticut was the first state in the nation to implement an Extreme Risk Protection Order (ERPO) law, in 1999. The Connecticut ERPO allows for anyone to warn the police about a person they believe is at risk of imminent harm to themselves or others and who may have access to firearms. If the police find there is a risk of imminent harm, they can ask a Superior Court judge to issue a “risk warrant” allowing the firearms to be removed and held for up to one year.

How the process works:
1. You must contact the local or state police and explain your concern
2. The police investigate
3. The police ask the court for a warrant to remove firearms
4. If the warrant is approved, police will remove all firearms and ammunition for the areas the judge approved.
5. The person's pistol permit and eligibility certificate will be revoked to prevent them from buying more firearms
6.The person has the right to a court hearing in 14 days. At the hearing the judge will decide if the firearms will be held by police for up to one year, transferred to someone else, or returned to the person. You would usually not have to attend the hearing if you contacted the police.
7. If the order is upheld the court will contact the Department of Emergency Services and Public Health Protection and the Department of Mental Health and Addiction Services.

Are you aware of any specific policies or procedures in place at your institution regarding ERPO?

Yes

No

Maybe

How likely would you say you are to call the police to request an ERPO for a patient or client at extreme risk of violence or suicide who you believe has access to firearms?

Extremely likely

Somewhat likely

Neither likely nor unlikely

Somewhat unlikely

Extremely unlikely

If you decide that you have a patient or client at extreme risk of violence or suicide, who you believe has access to firearms, how would you most likely report an ERPO?

I would call law enforcement myself

I would want a family member to call

I would not ever use this mechanism

I am not sure. Tell us why:

How often do you estimate you encounter a patient or client that is at extreme risk of violence or suicide, who you believe has access to firearms, and who you would consider for an ERPO?

Daily

Monthly

Weekly

A few times per year

Never

When you have a patient or client at risk of suicide, how often do you counsel them about lethal means?

Always

Most of the time

Some of the time

Rarely/never

If you discuss lethal means, do you ask about access to firearms?

Always

Most of the time

Some of the time

Rarely/never

What is your comfort level in discussing firearms with patients or clients who you believe are at risk of suicide?

Comfortable

Somewhat comfortable

Somewhat unfomfortable

Unformfortable

Have you ever used an extreme risk protection order?

Yes

No

How many times?

Did you find the outcome was helpful?

Definitely yes

Probably yes

Might or might not

Probably not

Definitely not

Would you be more or less likely to file a petition for an ERPO if you did not have to involve the police directly (e.g., you could petition the court yourself or you could contact a social worker to petition the court)?

More likely

The same

Less Likely

Other

What barrier(s) prevent you from using the ERPO law in Connecticut? Check all that apply

Not enough time to make the call and any follow up

Not a billable service

It may negatively affect my relationship with the patient

I don't think clinical providers should use ERPO mechanism

I don't feel comfortable involving the police in patient care

[ ] Other

What tools would help you file an ERPO? Check all that apply

Training on ERPO

Consultation with legal expert

A trained coordinator at my institution to call the police and follow through

If attendance at a hearing is required, allow participation remotely

A specific internal policy that my institution had implemented

A specific trigger in an informatics database when I entered information about a patient

[ ] Other

Would any of these diagnoses change your likelihood to file an ERPO?

	Click to write Column 1		
	More Likely	Less Likely	No Difference
Person has a history of diagnosis of depression	☐	☐	☐

	Click to write Column 1		
	More Likely	Less Likely	No Difference
Person has a history of diagnosis of psychosis	☐	☐	☐
Person has a history of traumatic brain injury	☐	☐	☐
Person has a history of a diagnosis of bipolar disorder	☐	☐	☐
Person has a history of a diagnosis of post-traumatic stress disorder	☐	☐	☐

Would a history of domestic violence or interpersonal violence change your likelihood to file an ERPO?

Make more likely

Make less likely

No difference

Would a history of incarceration change your likelihood to file an ERPO?

Make more likely

Make less likely

No difference

Please feel free to tell us anything at all
